# Multidimensional assessment of Juvenile Sjögren disease

**DOI:** 10.1007/s10067-025-07486-w

**Published:** 2025-05-30

**Authors:** Şeyma Türkmen, Taner Coşkuner, Betül Sözeri

**Affiliations:** Department of Pediatric Rheumatology, Ümraniye Training and Research Hospital, İstanbul, Turkey

**Keywords:** Juvenile Sjögren’s disease, Multidimensional approach, Nailfold videocapillaroscopy, Parotid gland ultrasound, Salivary gland biopsy, Schirmer test

## Abstract

**Objectives:**

This study aims to provide a multidimensional assessment of juvenile Sjögren’s disease (jSjD), focusing on diagnostic and management processes through various instrumental tests.

**Methods:**

A cross-sectional and retrospective study was conducted on 21 jSjD patients diagnosed between June 2016 and September 2023. In addition, 37 healthy children were included only for comparison of nailfold videocapillaroscopy (NVC) findings. Clinical data, parotid gland ultrasound (US), NVC, salivary gland biopsy, and Schirmer test results were analyzed.

**Results:**

Positive correlations were found between parotitis scores in parotid gland US and bizarre capillary, neoangiogenesis, and microhemorrhage scores in NVC (r = 0.35, 0.32, 0.52; p < 0.05). Diagnostic delay was associated with higher parotitis frequency and scores in US (p = 0.025) and increased dilated capillaries in NVC (p < 0.05). Clinically evident parotitis correlated with higher parotitis frequency in US (p = 0.009) and higher parotitis scores (r = 0.480, p = 0.051). Higher ESSDAI scores at diagnosis correlated with increased parotitis frequency and scores in US (p = 0.001), with higher scores in patients with high disease activity (p < 0.05). ENA-positive patients had higher parotitis scores in US (p = 0.022) and increased cross capillaries in NVC (p = 0.024). Capillary density was lower in the jSjD group compared to healthy controls (p = 0.021), with healthy children having higher median capillary density (p = 0.005). Dilated, bushy, bizarre capillaries, and microhemorrhages were significantly higher in the jSjD group (p < 0.001).

**Conclusion:**

Early and accurate diagnosis is crucial for managing jSjD. The integration of US and NVC provides a comprehensive framework for detecting glandular and microvascular abnormalities, emphasizing the need for multidimensional approach.

**Table:** 

***Key Points*** • *This study provides a multidimensional and comprehensive assessment of Juvenile Sjögren's disease (jSjD) by combining various instrumental tests used in the diagnosis and monitoring of Sjögren's disease.* • *It is the first study in the literature to demonstrate the connection between glandular and microvascular abnormalities in jSjD patients.* • *The comparison of nailfold videocapillaroscopy findings in healthy children highlights the pronounced microvascular abnormalities present in jSjD patients.* • *The results emphasize the importance of early diagnosis and intervention, offering critical insights into the systemic effects and clinical progression of the disease.*

## Introduction

Juvenile Sjögren’s disease (jSjD) is a rare chronic autoimmune disorder in children, posing significant diagnostic and treatment challenges. While primary Sjögren’s disease (pSjD) is more common in adult women, it also affects children, with symptoms appearing before age 18 [1–4]. Childhood-onset Sjögren’s disease accounts for about 1% of all cases [1].

The clinical presentation of jSjD differs from adults, with more prevalent glandular and systemic symptoms. Children often experience recurrent parotitis, xerostomia, and xerophthalmia [5, 6]. Extra-glandular manifestations include anemia, leukopenia, thrombocytopenia, lymphadenopathy, musculoskeletal involvement, interstitial lung disease, renal and hepatic disorders, vasculitis, central nervous system (CNS) involvement, and Raynaud’s phenomenon (RP) [7, 8]. There’s also a heightened risk of lymphoma [1, 7]. Autoantibodies like Antinuclear antibodies (ANA), anti-SSA (Ro), anti-SSB (La), and Rheumatoid factor (RF) are common, with decreased complement levels and elevated acute phase response in active disease [7, 9].

The American-European Consensus Group (AECG) and the American College of Rheumatology/European League Against Rheumatism (ACR/EULAR) criteria are used for classification, though they are more adult-focused [10, 11]. Proposed pediatric criteria from 1999 lack sensitivity [12–14]. The EULAR Sjögren’s Syndrome Disease Activity Index (ESSDAI) is crucial for assessing disease activity [15, 16].

Non-invasive diagnostic tools such as salivary gland ultrasound and nailfold videocapillaroscopy (NVC) are vital for diagnosing and monitoring jSjD [2, 4, 7, 8]. Parotid gland ultrasound (US) helps determine glandular involvement, using findings like glandular heterogeneity, hypoechoic areas, and border disruptions [2, 4]. Various scoring systems exist to evaluate salivary gland pathology [17]. The Outcome Measures in Rheumatology (OMERACT) ultrasound working group has developed a gray-scale semi-quantitative consensus scoring system for major salivary glands [18, 19]. They later created a scoring system based on Doppler activity [20]. However, distinguishing between normal and pathological vascularization, as well as the challenges posed by individual differences, can present some problems [20]. Additionally, pathological changes in pSjD are closely related to morphological changes, making B-mode the primary ultrasound mode [21].

Nailfold videocapillaroscopy evaluates microvascular abnormalities, revealing changes like capillary morphology abnormalities, decreased density, and neoangiogenesis in Sjögren’s patients, though a specific pattern for pSjD is undefined [7, 8]. Although neither method is included in the classification criteria, their non-invasive and reproducible nature makes them highly beneficial for the early diagnosis and follow-up of jSjD [2, 7].

Salivary gland biopsy remains the gold standard for diagnosing pSjD, showing lymphocytic sialadenitis and focal lymphocytic infiltration. Schirmer’s test, assessing tear production, is crucial for detecting tear insufficiency in jSjD [3, 22].

This study aims to comprehensively assess jSjD, focusing on its diagnosis and management. By providing crucial data on the clinical course and systemic effects of jSjD, it will lay the foundation for future research and clinical practice. Given the scarcity of childhood studies, this will be the first to offer a multidimensional assessment of jSjD.

## Materials and methods

### Patients

This cross-sectional and retrospective study included 21 jSjD patients followed for at least six months and 37 healthy children at the Pediatric Rheumatology Clinic, University of Health Sciences Ümraniye Training and Research Hospital, between June 2016 and September 2023. Healthy controls were included exclusively for comparison of NVC findings and did not undergo any other clinical or instrumental evaluations. The study adhered to the Declaration of Helsinki and was approved by the ethics committee of the University of Health Sciences Ümraniye Training and Research Hospital (Ethics committee no: B.10.1.TKH.4.34.H.GP.0.01/227). Written informed consent was obtained from the legal guardians of all patients and from patients aged 12 or older.

Inclusion criteria for the patient group were:

- Diagnosis of primary Sjögren’s disease before the age of 18.
- Meeting the AECG criteria (10) and/or the proposed 1999 jSjD criteria (12) for those diagnosed before 2017.
- Meeting the ACR/EULAR criteria (11) for those diagnosed in 2017 and beyond.
- Having a follow-up period of at least six months.

To ensure diagnostic consistency across the cohort, all patients were retrospectively re-evaluated according to the ACR/EULAR classification criteria (11).

Exclusion criteria for the patient group at the last visit for NVC assessment included acute trauma to the fingers, past surgeries, or a history of manicure within the last month.

Inclusion criteria for the healthy group were:

- No systemic chronic disease.
- No infectious/inflammatory disease or congenital anomaly of the fingers.
- No history of acute trauma, previous surgery, or manicure within the last month related to the fingers.

At the time of diagnosis, all patients had undergone standard laboratory investigations including autoantibodies, inflammatory markers, and blood counts, as well as salivary gland biopsy and Schirmer test.

At the last visit, a structured evaluation was performed specifically for this study, including physical examination, ESSDAI scoring, parotid gland US, and NVC.

These time points have been clearly differentiated in all analyses.

Demographic data, clinical features, laboratory results at diagnosis, salivary gland biopsy and Chisholm Mason classification results [23], Schirmer test results, treatment regimens during follow-up, and treatment responses were retrospectively recorded from the files.

Demographic data included age, gender, date of symptom onset, date of diagnosis, date of last visit, follow-up duration, comorbid diseases, consanguinity in the family, and family history of rheumatic diseases. Diagnostic delay was defined as the time interval between symptom onset and the date of diagnosis.

Clinical features at diagnosis included constitutional symptoms; glandular and ophthalmologic findings (xerostomia, xerophthalmia, clinically evident parotitis including recurrent parotitis); RP; skin and/or mucosal involvement; musculoskeletal involvement; renal findings; neurological findings; gastrointestinal findings; cardiovascular system findings; pulmonary findings; ESSDAI score and disease activity at diagnosis.

Systemic involvement was defined based on the ESSDAI scoring system [15] whenever applicable. For systems not covered by ESSDAI, definitions were based on current clinical practice:

- Constitutional involvement: Presence of fever (> 38 °C), unexplained weight loss, and/or fatigue, lymphadenopathy as documented in medical records.
- Skin and mucosal involvement: Cutaneous vasculitis, palpable purpura, or recurrent oral ulcers observed during physical examination or confirmed by biopsy.
- Musculoskeletal involvement: Arthralgia, arthritis, or myalgia confirmed by physical examination; cases of myositis diagnosed by elevated muscle enzymes, MRI, or electromyography were also included.
- Renal involvement: Active renal pathology including proteinuria (> 0.5 g/day), hematuria, reduced glomerular filtration rate (< 60 mL/min), or presence of renal tubular acidosis; chronic or damage-related findings were excluded.
- Neurological involvement: Central and/or peripheral nervous system findings. Central symptoms included headache, seizures, or cognitive dysfunction supported by neurological examination or imaging. Peripheral findings included neuropathy confirmed by clinical or electrophysiological assessment.
- Gastrointestinal involvement: Persistent gastrointestinal symptoms such as abdominal pain, diarrhea, or nausea not attributable to infection or treatment side effects and evaluated in clinical context.
- Hematological involvement: Presence of autoimmune cytopenias, including anemia (Hb < 11 g/dL), leukopenia (< 4000/mm^3^), lymphopenia (< 1500/mm^3^), or thrombocytopenia (< 150.000/mm^3^), excluding other causes such as iron/vitamin deficiency or medication effects.
- Cardiac involvement: Myocarditis, pericarditis, or conduction abnormalities confirmed by clinical, electrocardiographic, or echocardiographic findings, when documented.
- Pulmonary involvement: Interstitial lung disease or small airway disease confirmed by chest imaging or pulmonary function tests at the time of diagnosis.

Laboratory findings at diagnosis included anemia (Hemoglobin < 11 g/dL), leukopenia (< 4000/mm3), lymphopenia (< 1500/mm3), thrombocytopenia (< 150.000/mm3), elevated C-reactive protein (CRP), elevated erythrocyte sedimentation rate (ESR), complement 3 and 4 (C3 and C4) levels, ANA positivity, titer and patterns, anti-double-stranded DNA (anti-ds DNA), Extractable Nuclear Antigens (ENA) panel (including anti-SSA (Ro) and anti-SSB (La), anti-Ribonucleoprotein (anti-RNP)), RF, and anti-cyclic citrullinated peptide (anti-CCP) results.

Clinically evident parotitis was defined as the presence of at least one of the following: pain, tenderness, or swelling in the parotid gland region. ANA assessed with indirect immunofluorescence (IIFA) on Hep-2/liver cells was considered positive at a titer of 1/80 or above at diagnosis. The Schirmer test was considered positive (abnormal) if tear production was ≤ 5 mm/5 min. Disease activity was measured with the ESSDAI. The ESSDAI’s 12 domains—constitutional, lymphadenopathy, glandular, articular, cutaneous, muscular, renal, pulmonary, peripheral nervous system, central nervous system, hematological, and biological—were assessed for each patient. Scores ranging from 0 to 4 were considered low disease activity, 5–13 as moderate, and 14 or above as high disease activity [15]. Complete remission was defined as the absence of significant clinical findings, an ESSDAI score of less than 5, no active glandular involvement (such as parotitis or marked xerostomia/xerophthalmia), and no evidence of active systemic findings (e.g., arthritis, renal or neurological findings). Partial remission was defined as an improvement in disease activity without achieving complete clinical resolution, characterized by the presence of mild systemic findings (e.g., fatigue, intermittent arthralgia) and/or partial improvement in glandular findings, despite an ESSDAI score remaining below 5. At the last visit, patients underwent a detailed physical examination, and their ESSDAI scores and disease activity levels were assessed by TC and recorded. Parotid gland ultrasonography was performed by a EULAR-certified operator (BS) and classified using the OMERACT scoring system [18, 19]. NVC was conducted by a trained and certified operator (ŞT), with findings recorded. Due to time constraints or missed appointments, some patients did not undergo parotid gland US and NVC. Patients who did not meet the eligibility criteria were not assessed with NVC at the last visit. NVC was also performed in healthy children by the same operator (ŞT) during a separate session, ensuring adherence to the standardized procedure. Although full blinding to diagnosis was not feasible in the clinical setting, each of the three pediatric rheumatologists (performing physical examination and ESSDAI assessment, parotid gland US, and NVC, respectively) conducted their evaluations independently and were unaware of the others’ findings to avoid inter-observer bias in the correlation analyses.

### Parotid gland ultrasound

Parotid gland US scans were performed using a LOGIQ e device equipped with a 9–15 MHz linear probe. Both parotid glands were scanned bilaterally in longitudinal and transverse planes. Only B-mode was used during the scans. The images were evaluated using the OMERACT gray-scale semi-quantitative consensus scoring system [18, 19], as follows:

- Grade 0: Normal parenchyma
- Grade 1: Mild heterogeneity without anechoic or hypoechoic areas and hyperechoic bands
- Grade 2: Moderate heterogeneity with focal anechoic or hypoechoic areas
- Grade 3: Severe heterogeneity with extensive anechoic or hypoechoic areas throughout the gland or a fibrotic gland.

### Nailfold videocapillaroscopy examination

Nailfold videocapillaroscopy examination was performed on jSjD patients and healthy controls by the single physician, using an optical probe with 200 × magnification and image analysis software (DinoCapture 2.0 Version 1.5.48).

Following the standard protocol, each patient waited in a room at 20–22 °C for at least 15 min before undergoing NVC. Four digital images were collected from a 2-mm area in the middle of eight nailfolds, excluding the thumbs.

The following capillaroscopic parameters were evaluated: capillary density (< 7 capillaries/mm considered abnormal), capillary enlargements (diameter 20–50 μm), giant capillaries (diameter ≥ 50 μm), microhemorrhages, and abnormal shapes (bushy capillaries, non-convex ends, capillaries crossing ≥ 3 times) [24, 25]. A semi-quantitative grading scale by Cutolo et al. was used to score these parameters (0, no change; 1, < 33% alteration; 2, 33–66% alteration; 3, > 66% alteration per linear millimeter) [26]. The average absolute number of capillaries per linear millimeter (capillary density) was calculated using all 32 images collected per individual [27]. If a"scleroderma pattern"was present, it was assigned according to the 2019 Fast Track algorithm by Smith et al. [28].

### Statistical analysis

Descriptive statistics were provided as mean, median, and interquartile range (IQR) for continuous variables, and as number and percentage for categorical variables. Spearman correlation was used for ordinal variables. The Chi-square and Fisher’s exact tests compared group distributions. The Mann–Whitney U and Kruskal–Wallis tests explored heterogeneity among categories. Linear regression analyzed the association between dependent and independent variables. Statistical analyses were conducted using IBM SPSS Statistics version 25.0, with significance set at p < 0.05.

## Results

### Demographic and clinical characteristics of patients

A total of 21 jSjD patients were evaluated. At the time of diagnosis, 19 patients (90.47%) met the ACR/EULAR classification criteria, while 2 patients (9.53%) were diagnosed based on the AECG and Proposed Pediatric Criteria due to the earlier diagnosis date. To ensure diagnostic consistency across the cohort, all patients were retrospectively re-evaluated and confirmed to fulfill the ACR/EULAR classification criteria. Additionally, 9.53% (n = 2) had mixed connective tissue disease (MCTD), and 4.76% (n = 1) had systemic lupus erythematosus, but jSjD symptoms were predominant.

Of the patients, 95.24% (n = 20) were female. Comorbid diseases were present in 14.28% (n = 3) (two with fibromyalgia, one with Familial Mediterranean Fever (FMF)). Consanguinity was present in 9.52% (n = 2), and 23.80% (n = 5) had a family history of rheumatic diseases (2 pSjD, 2 Rheumatoid Arthritis, 1 FMF).

The median age at symptom onset was 12.7 years (IQR: 11.4–15.3) and at diagnosis was 13 years (IQR: 12–16). The median diagnostic delay was 12.65 months (IQR: 5.75–17.71), and the median follow-up duration was 39 months (IQR: 16–64).

At diagnosis, xerostomia and xerophthalmia were observed in 71.42% (n = 15) and 76.19% (n = 16) of patients, respectively. Clinical signs of parotitis were found in 28.57% (n = 6) and recurrent parotitis in 9.52% (n = 2).

All but one patient had at least one extra-glandular manifestation at diagnosis. Constitutional symptoms were present in 33.33% (n = 7), RP in 19.04% (n = 4), skin and mucosal involvement in 33.33% (n = 7), musculoskeletal involvement in 76.19% (n = 16), renal involvement in 19.04% (n = 4), neurological involvement in 14.28% (n = 3), gastrointestinal involvement in 14.28% (n = 3), and hematological involvement in 19.04% (n = 4). No patients had cardiovascular or pulmonary involvement.

The median ESSDAI score at diagnosis was 6 (IQR: 0–9) and 0 (IQR: 0–0) at the last visit. At diagnosis, 14.28% (n = 3) had high, 47.62% (n = 10) had moderate, and 38.1% (n = 8) had low disease activity. By the last visit, ESSDAI scores had decreased in all patients, with all achieving low disease activity.

At diagnosis, 85.71% (n = 18) of patients were ANA positive, and 33.33% (n = 7) had a positive ENA profile (6 with anti-SSA, 2 with anti-SSB, and 3 with anti-RNP). RF was positive in 19.04% (n = 4) and CCP in one patient. Elevated CRP was found in 23.80% (n = 5) and elevated ESR in 19.04% (n = 4).

The conventional treatments were hydroxychloroquine (95.23%, n = 20), methotrexate (76.19%, n = 16), corticosteroids (38.09%, n = 8), mycophenolate mofetil (38.09%, n = 8), and azathioprine (4.76%, n = 1). Rituximab was given to 19.04% (n = 4) and abatacept to 4.76% (n = 1). At the last visit, 90.48% (n = 19) were in complete remission and 9.52% (n = 2) in partial remission. Treatment details are summarized descriptively as supportive information.

The demographic and clinical characteristics of the juvenile Sjögren’s patients are summarized in Table 1.

**Table 1:** Demographic and Clinical Characteristics of Juvenile Sjögren’s Disease Patients

Characteristic	Value
Total number of patients	21
Met ACR/EULAR 2016 criteria	90.47% (n = 19)
Met AECG and Pediatric criteria	9.53% (n = 2)
Overlapping MCTD	9.53% (n = 2)
Overlapping SLE	4.76% (n = 1)
Female patients	95.24% (n = 20)
Comorbid diseases	14.28% (n = 3)
- Fibromyalgia	66.66% (n = 2)
- FMF	33.33% (n = 1)
Familial consanguinity	9.52% (n = 2)
Family history of rheumatic diseases	23.80% (n = 5)
- pSjD	40% (n = 2)
- RA	40% (n = 2)
- FMF	20% (n = 1)
Median age at symptom onset	12.7 years (IQR: 11.4–15.3)
Median age at diagnosis	13 years (IQR: 12–16)
Median diagnostic delay	12.65 months (IQR: 5.75–17.71)
Median follow-up duration	39 months (IQR: 16–64)
Xerostomia at diagnosis	71.42% (n = 15)
Xerophthalmia at diagnosis	76.19% (n = 16)
Clinical parotitis at diagnosis	28.57% (n = 6)
Recurrent parotitis	9.52% (n = 2)
Patients with extraglandular manifestations	95.24% (n = 20)
Constitutional symptoms	33.33% (n = 7)
RP	19.04% (n = 4)
Skin and mucosal involvement	33.33% (n = 7)
- Oral ulcer	4.76% (n = 1)
- Malar rash	23.80% (n = 5)
- Facial erythema	4.76% (n = 1)
- Photosensitivity	19.04% (n = 4)
Musculoskeletal involvement	76.19% (n = 16)
- Arthralgia	76.19% (n = 16)
- Arthritis	52.38% (n = 11)
- Myalgia	42.85% (n = 9)
Renal involvement	19.04% (n = 4)
- Proteinuria	50% (n = 2)
- Hematuria	50% (n = 2)
Neurological involvement	14.28% (n = 3)
- Acute confusional state	33.33% (n = 1)
- Headache	66.66% (n = 2)
Gastrointestinal involvement	14.28% (n = 3)
- Difficulty in swallowing	33.33% (n = 1)
- Gastroesophageal reflux	33.33% (n = 1)
- Gastritis	33.33% (n = 1)
- Constipation	33.33% (n = 1)
Hematological involvement	19.04% (n = 4)
- Anemia	75% (n = 3)
- Lymphopenia	75% (n = 3)
Median ESSDAI score at diagnosis	6 (IQR: 0–9)
Median ESSDAI score at last visit	0 (IQR: 0–0)
High disease activity (at diagnosis)	14.28% (n = 3)
Moderate disease activity	47.62% (n = 10)
Low disease activity	38.1% (n = 8)
ANA positive	85.71% (n = 18)
ENA profile positive	33.33% (n = 7)
- Anti-SSA	85.71% (n = 6)
- Anti-SSB	28.57% (n = 2)
- Anti-RNP	42.85% (n = 3)
RF positive	19.04% (n = 4)
CCP positive	4.76% (n = 1)
Elevated CRP (at diagnosis)	23.80% (n = 5)
Elevated ESR (at diagnosis)	19.04% (n = 4)
Hydroxychloroquine treatment	95.23% (n = 20)
Methotrexate treatment	76.19% (n = 16)
Corticosteroid treatment	38.09% (n = 8)
Mycophenolate mofetil treatment	38.09% (n = 8)
Azathioprine treatment	4.76% (n = 1)
Rituximab treatment	19.04% (n = 4)
Abatacept treatment	4.76% (n = 1)
Complete remission	90.48% (n = 19)
Partial remission	9.52% (n = 2)

*ACR* american college of rheumatology, *EULAR* european league against rheumatism, *AECG* american-european consensus group, *MCTD* mixed connective tissue disease, *SLE* systemic lupus erythematosus, *FMF* familial mediterranean fever, *pSjD* primary sjögren’s disease, *RA* rheumatoid arthritis, *IQR* interquartile range, *SSA* sjögren’s syndrome-related antigen A, *SSB* sjögren’s syndrome-related antigen B, *RNP* ribonucleoprotein, *RF* rheumatoid factor, *CCP* cyclic citrullinated peptide, *CRP* c-reactive protein, *ESR* erythrocyte sedimentation rate, *ENA* extractable nuclear antigen, *ESSDAI* eular sjögren’s syndrome disease activity index

### Parotid gland ultrasound results

Parotid gland ultrasound was performed on 80.95% (n = 17) of patients at the last visit. Among these, 58.82% (n = 10) had ultrasound findings consistent with parotitis. According to the OMERACT scoring system, 41.17% (n = 7) were grade 0, 35.29% (n = 6) were grade 1, and 23.52% (n = 4) were grade 2. No patients had grade 3 parotitis.

In this study, increased diagnostic delay correlated with higher frequency and scores of parotitis observed in parotid gland US (p = 0.025 for each). Patients with clinically evident parotitis at baseline had a significantly higher frequency of parotitis in US (p = 0.009). Ultrasonographic parotitis scores were moderately higher in these patients, with borderline statistical significance (r: 0.480, p = 0.051).

As the ESSDAI score at diagnosis increased, both the frequency of parotitis observed in parotid gland US and the parotitis score increased (p = 0.001 for each). Patients with high disease activity had significantly higher frequencies of parotitis in US and higher parotitis scores (p = 0.015 and 0.032, respectively).

Patients with ENA positivity had significantly higher parotitis scores in US (p = 0.022).

### Nailfold videocapillaroscopy results

Nailfold videocapillaroscopy was performed on 61.9% (n = 13) of patients at the last visit, with a median age of 18 years (IQR 14–20). Minor capillary morphology abnormalities were observed in all patients, but no scleroderma pattern was detected. All patients exhibited at least two minor morphological abnormalities. The median capillary density was 6.625 (IQR 6.375–7.25), with 61.53% (n = 8) having low capillary density (< 7). Increased tortuosity was found in 69.23% (n = 9), cross capillaries in 76.92% (n = 10), and dilated capillaries in 23.07% (n = 3); no giant capillaries were detected. Capillary meandering was observed in 46.15% (n = 6), bushy capillaries in 38.46% (n = 5), and bizarre capillaries with neoangiogenesis in 61.53% (n = 8). Avascular areas were found in 23.07% (n = 3), and microhemorrhages in 53.84% (n = 7). Only one patient had RP, and this patient exhibited capillary dilatation and neoangiogenesis with bushy and bizarre capillaries.

As the diagnostic delay increased, the frequency of dilated capillaries also increased significantly, with p-values of 0.05, 0.021, and 0.0104 at delays of 12, 18, and 24 months, respectively.

A significant association (p = 0.024) was observed between ENA positivity and an increase in cross capillaries in the NVC findings.

**Fig. 1:**
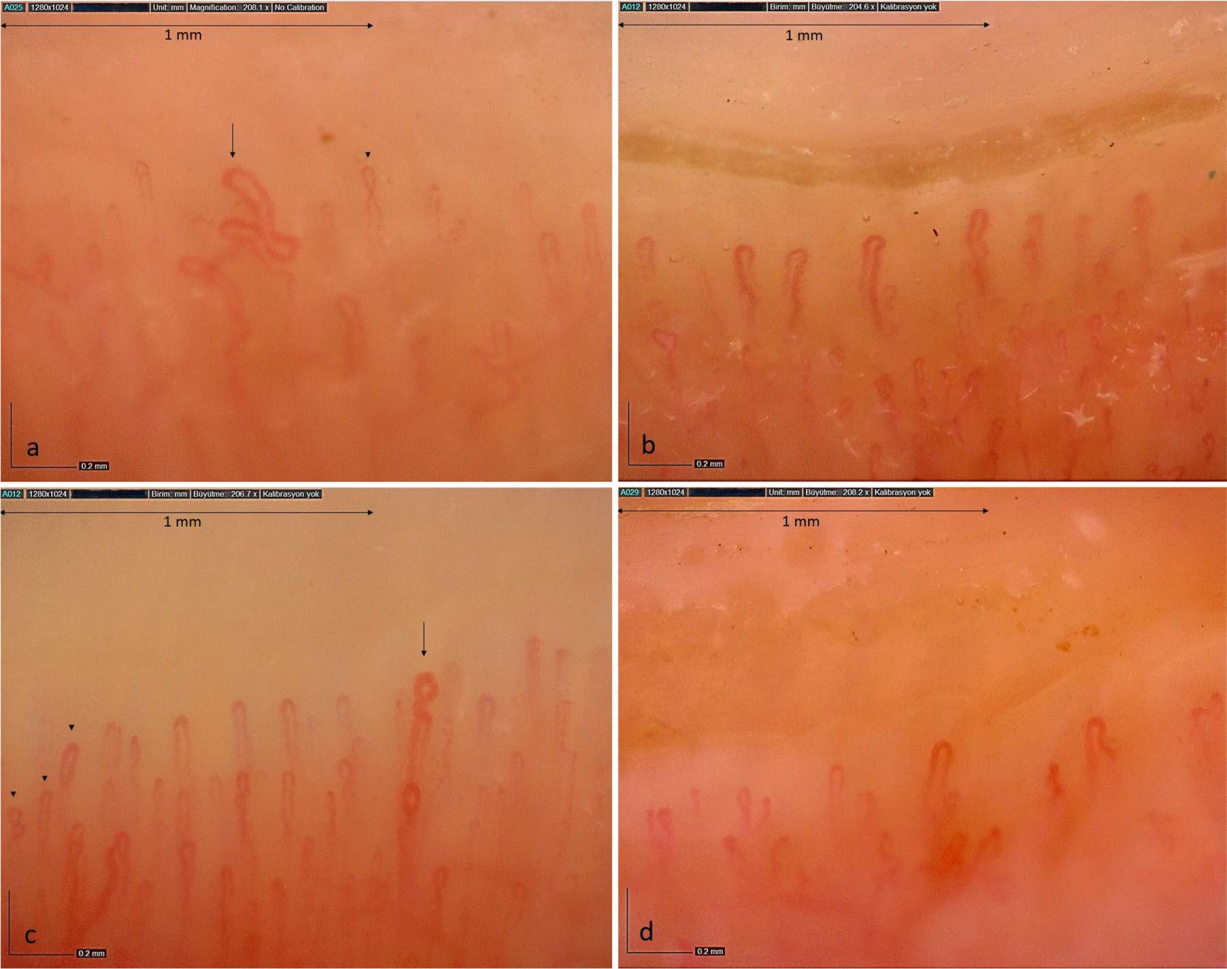
Examples of nailfold videocapillaroscopy images of four patients with juvenile Sjögren's syndrome (magnification 200x); **a**. One bizarre capillary (arrow) indicating neoangiogenesis and a cross capillary (arrowhead), along with reduced capillary density (5 capillaries/mm) **b**. Reduced capillary density (5 capillaries/mm) **c**. One dilated capillary (arrow) and cross capillaries (arrowheads) with normal capillary density **d**. Reduced capillary density (4 capillaries/mm)

Nailfold videocapillaroscopy images of four jSjD patients are presented in Fig. 1.

In the control group (n = 37, median age 16 years, IQR 15–17), NVC was completely normal in 18.92% (n = 7), while minor abnormalities—mostly isolated increases in tortuous or cross capillaries—were observed in the rest. No healthy child had dilated, bizarre, or giant capillaries, nor microhemorrhages. Neoangiogenesis was observed in 5.4% (n = 2), and avascular areas in 8.1% (n = 3)﻿.

Capillary density was significantly lower in the jSjD group compared to healthy controls (p = 0.021). The median capillary density in controls was 7.5 (IQR 7–8), significantly higher than in the jSjD group (p = 0.0051). Dilated capillaries (p = 0.015), as well as bushy, meandering, bizarre capillaries, neoangiogenesis, and microhemorrhages were significantly more frequent in the jSjD group (p = 0.003, 0.001, and p < 0.001 for others).

In the control group, NVC was performed on 37 healthy children with a median age of 16 years (IQR 15–17). There was no significant difference in median age between the patient and healthy groups (p = 0.455). NVC was completely normal in 18.92% (n = 7) of healthy children, while minor morphological abnormalities were detected in the others. Most abnormalities (93.3%) were isolated increases in cross and tortuous capillaries. Only one child each had bushy capillaries and combined morphological abnormalities with capillary meandering. No healthy child exhibited dilated, giant, bizarre capillaries, or microhemorrhages; however, neoangiogenesis was found in 5.4% (n = 2) and isolated avascular areas in 8.1% (n = 3).

Low capillary density was found in 21.62% (n = 8) of healthy children, but the capillary density in the jSjD group was significantly lower (p = 0.021). The median capillary density in healthy children was 7.5 (IQR: 7–8), significantly higher than in the jSjD group (p = 0.0051). Dilated capillaries were significantly more common in the jSjD group (p = 0.015). Additionally, bushy, meandering, bizarre capillaries, neoangiogenesis, and microhemorrhages were significantly higher in the jSjD group (p = 0.003, 0.001, and p < 0.001 for the others).

### Salivary gland biopsy results

Salivary gland biopsy was performed on all patients at diagnosis. According to the Chisholm-Mason classification, 14.28% (n = 3) were Grade 0, 33.33% (n = 7) were Grade 2, 23.80% (n = 5) were Grade 3, and 28.57% (n = 6) were Grade 4.

As the ESSDAI scores at diagnosis increased, biopsy grades also increased; however, the correlation was weak to moderate and not statistically significant (r = 0.326, p = 0.149).

### Schirmer test results

The Schirmer test was performed on all patients at diagnosis, with 33.33% (n = 7) testing positive.

As the ESSDAI scores at diagnosis increased, the rate of positive Schirmer test results showed a weak correlation but was not statistically significant (r = 0.285, p = 0.207). Additionally, positive Schirmer test results were more frequent in patients with moderate and/or high disease activity according to ESSDAI scores, but this association was not statistically significant (p = 0.232).

### Associations between instrumental tests

**Fig. 2:**
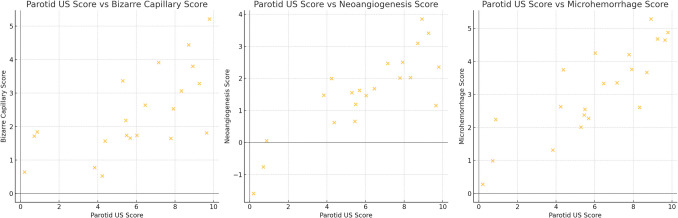
Correlations between parotitis scores in parotid gland ultrasound and bizarre capillary scores, neoangiogenesis scores, and microhemorrhage scores in nailfold videocapillaroscopy

Positive correlations were found between parotitis scores in parotid gland US and bizarre capillary scores, neoangiogenesis scores, and microhemorrhage scores in NVC (r = 0.35, 0.32, 0.52 and p = 0.04, 0.046, 0.031, respectively) (Fig. 2).

The instrumental test results and associations between the clinical findings of juvenile Sjögren patients are presented in Table 2.

**Table 2:** Instrumental Test Results and Associations Between The Clinical Findings of Juvenile Sjögren Patients

Instrumental Test	Value
Parotid Gland Ultrasound	
Patients evaluated	80.95% (n = 17)
Findings consistent with parotitis	58.82% (n = 10)
OMERACT scoring	
- Grade 0	41.17% (n = 7)
- Grade 1	35.29% (n = 6)
- Grade 2	23.52% (n = 4)
- Grade 3	0% (n = 0)
Nailfold Videocapillaroscopy	
Patients evaluated	61.9% (n = 13)
Median age of patients	18 years (IQR: 14–20)
Minor capillary morphology abnormalities	100% (n = 13)
Mean capillary density	6.625 (IQR: 6.375–7.25)
Low capillary density (< 7/mm)	61.53% (n = 8)
Tortuous capillaries	69.23% (n = 9)
Crossed capillaries	76.92% (n = 10)
Dilated capillaries	23.07% (n = 3)
Giant capillaries	0% (n = 0)
Meandering capillaries	46.15% (n = 6)
Bushy capillaries	38.46% (n = 5)
Bizarre capillaries	61.53% (n = 8)
Avascular areas	23.07% (n = 3)
Microhemorrhages	53.84% (n = 7)
Salivary Gland Biopsy	
Patients evaluated	100% (n = 21)
Chisholm Mason classification	
- Grade 0	14.28% (n = 3)
- Grade 1	0% (n = 0)
- Grade 2	33.33% (n = 7)
- Grade 3	23.80% (n = 5)
- Grade 4	28.57% (n = 6)
Schirmer Test	
Patients evaluated	100% (n = 21)
Positive Schirmer test	33.33% (n = 7)
Correlations and Associations	
Ultrasonographic parotitis vs. diagnosis delay	p = 0.025
Clinical parotitis vs. ultrasonographic parotitis	p = 0.009
Diagnosis delay	
- NVC dilated capillaries (12 months)	p = 0.050
- NVC dilated capillaries (18 months)	p = 0.021
- NVC dilated capillaries (24 months)	p = 0.010
ESSDAI score at diagnosis vs. ultrasonographic parotitis	p = 0.001
ESSDAI score at diagnosis vs. salivary gland biopsy	r = 0.326, p = 0.149
ESSDAI score at diagnosis vs. Schirmer test	r = 0.285, p = 0.207
Moderate-to-high disease activity vs. Schirmer test	p = 0.232
Ultrasonographic parotitis score	
- Bizarre capillaries	r = 0.35, p < 0.05
- Neoangiogenesis	r = 0.32, p < 0.05
- Microhemorrhages	r = 0.52, p < 0.05

*OMERACT* outcome measures in rheumatology, *NVC* nailfold videocapillaroscopy, *IQR* interquartile range, *mm* millimeter, *ESSDAI* EULAR sjögren’s syndrome disease activity index

The comparison of NVC findings of juvenile Sjögren patients with healthy controls is presented in Table 3.

**Table 3:** Nailfold Videocapillaroscopy Findings in Juvenile Sjögren’s Disease Patients Compared to Healthy Controls

Characteristic	jSjD Patients (n = 13)	Healthy Controls (n = 37)	Comparison
Median age	18 years (IQR: 14–20)	16 years (IQR: 15–17)	p = 0.455
Completely normal NVC	0% (n = 0)	18.92% (n = 7)	p = 0.168
Minor morphological abnormalities	100% (n = 13)	81.08% (n = 30)	p = 0.168
Dilated capillaries	23.07% (n = 3)	0% (n = 0)	p = 0.015
Giant capillaries	0% (n = 0)	0% (n = 0)	
Bushy capillaries	38.46% (n = 5)	2.7% (n = 1)	p = 0.003
Meandering capillaries	46.15% (n = 6)	2.7% (n = 1)	p = 0.001
Bizarre capillaries	61.53% (n = 8)	0% (n = 0)	p < 0.001
Neoangiogenesis	61.53% (n = 8)	5.4% (n = 2)	p < 0.001
Microhemorrhages	53.84% (n = 7)	0% (n = 0)	p < 0.001
Avascular areas	23.07% (n = 3)	8.1% (n = 3)	p = 0.173
Low capillary density (< 7/mm)	61.53% (n = 8)	21.62% (n = 8)	p = 0.021
Median capillary density	6.625 (IQR: 6.375–7.25)	7.5 (IQR: 7–8)	p = 0.005

*jSjD* juvenile sjögren’s disease, *NVC* nailfold videocapillaroscopy, *IQR* interquartile range, *mm* millimeter

## Discussion

This study presents a multifaceted and comprehensive assessment of jSjD. Combining various instrumental tests used in the diagnosis and monitoring of Sjögren’s disease, this study is, to our knowledge, the first of its kind in the literature. It makes a significant contribution to the literature as the first study to demonstrate the connection between glandular and microvascular abnormalities. The comparison of NVC findings in healthy children highlights the pronounced microvascular abnormalities present in jSjD patients. The results provide critical insights into the systemic effects and clinical progression of the disease, underscoring the importance of early diagnosis and intervention. These findings suggest that prolonged diagnostic delay may have persistent effects on microvascular structure, which are detectable even after achieving low disease activity at follow-up. Similarly, the significant correlation between diagnostic delay and parotitis scores on ultrasound—despite low disease activity at follow-up—may also reflect a lasting impact of delayed diagnosis on glandular involvement. This study sets a new standard for understanding jSjD, emphasizing the necessity of a multidisciplinary approach in managing and diagnosing this rare and complex autoimmune disease.

Our findings reveal significant differences in glandular and extra-glandular manifestations in jSjD. Diagnostic delay increased both the frequency and severity of parotitis observed in parotid gland US, highlighting US’s sensitivity in detecting glandular involvement. Numerous publications, including both pediatric and adult studies, support the utility of salivary gland US in evaluating Sjögren’s disease [1, 2, 16, 29–33]. In 2020, Krumrey-Langkammerer et al. reported parenchymal heterogeneity and hypoechoic areas as main pathological changes in jSjD on US, with no correlation between disease duration and US findings [4].

Another finding was the higher frequency of parotitis detected by US in patients with clinically evident parotitis at diagnosis. In 2014, Nieto-González et al. reported typical ultrasound findings in children with recurrent parotitis [32]. Our study also showed that higher ESSDAI scores at diagnosis correlated with increased frequency and severity of parotitis in US, particularly in patients with high disease activity. These correlations suggest that higher disease activity at diagnosis is associated with more severe glandular involvement, consistent with Hammenfors et al. (2020) [2].

The higher parotitis scores in US in ENA-positive patients suggest a link between serological markers and glandular pathology. Hammenfors et al. (2020) also reported a association between autoantibody positivity and salivary gland US results in children [2].

In our study, no scleroderma pattern was detected in NVC in any jSjD patients, but all exhibited minor morphological abnormalities. Capillary density was significantly lower compared to the healthy group, while dilated, bushy, meandering, bizarre capillaries, neoangiogenesis, and microhemorrhages were significantly higher in the jSjD group.

Our results indicate that diagnostic delay affects NVC findings. As the diagnostic delay increased, the frequency of dilated capillaries in NVC also increased, highlighting the importance of early diagnosis for microvascular health. More frequent detection of cross capillaries in ENA-positive patients suggests that autoantibody positivity impacts microvascular health as well as glandular health.

Pediatric studies on NVC in Sjögren’s disease are scarce, and the current literature is largely derived from adult cohorts. A 2020 review by Melsens et al., based predominantly on studies in adult populations, highlighted the lack of studies on NVC in pSjD, with existing data insufficiently distinguishing pSjD from healthy controls [8]. Lercara et al. found higher rates of abnormal NVC findings in jSjD, with joint involvement linked to low capillary density and microhemorrhages related to low C3 levels [7]. Capobianco et al. reported scleroderma-like patterns in a small but significant portion of adult pSjD, suggesting these could be manifestations of systemic vasculitis [34]. Riccieri et al. associated NVC abnormalities with anti-endothelial cell antibodies, indicating potential endothelial damage in adult pSjD patients [35]. Corominas et al. reported non-specific capillaroscopic abnormalities in 27.2% and scleroderma-like patterns in 10.2% of adult pSjD patients, with no association with anti-SSA/Ro or anti-SSB/La antibodies. They found more dilated capillaries in pSjD associated with RP [36]. Hysa et al. noted no specific NVC patterns for adult pSjD patients but reported lower capillary density in those with RP [37]. Since only one of our NVC patients had RP, we could not perform a statistical analysis.

Our findings on the correlation between parotid gland US and NVC scores are novel. Positive correlations were found between parotitis US scores and NVC scores, particularly for bizarre capillaries, neoangiogenesis, and microhemorrhages. This is the first study to demonstrate the connection between glandular and microvascular abnormalities, significantly contributing to the literature.

One strength of this study is the integration of clinical, ultrasonographic, and capillaroscopic data, providing a comprehensive understanding of jSjD. Physical examinations, US, and NVC evaluations were conducted blindly by different pediatric rheumatologists, enhancing objectivity and reliability. This study is the first to combine various instrumental tests for diagnosing and monitoring jSjD, highlighting the necessity of classification criteria for children and the potential inclusion of salivary gland US and NVC.

However, our study has limitations. Due to real-life constraints such as missed appointments and cooperation issues in pediatric patients, not all instrumental evaluations could be performed in every case. However, all patients underwent salivary gland biopsy and Schirmer test at diagnosis, and the majority received at least three different instrumental assessments, supporting the multidimensional nature of our approach. The retrospective design may lead to incomplete clinical and laboratory data, limiting generalizability. The small sample size and single-center nature also limit applicability to a broader population. Procedures performed by single operators (one for US and one for NVC) may introduce bias. The lack of initial US and NVC data restricts understanding changes with treatment. Additionally, the absence of follow-up data precludes statistical analysis of treatment responses. Some findings were insufficient for statistical analysis due to the small number of patients. Future multicenter, prospective studies with larger samples and standardized diagnostic criteria are needed to validate and expand our findings.

In conclusion, this study emphasizes the importance of early and accurate diagnosis in managing jSjD. The integration of US and NVC provides a comprehensive framework for detecting glandular and microvascular abnormalities. This study enhances our understanding of jSjD and lays the foundation for future research and clinical practice, ultimately aiming to improve patient outcomes and quality of life.

## Data Availability

The data sets analyzed during the current study are available from the corresponding author on reasonable request.
